# Retraction: Preparation of NiFe_2_O_4_@MIL-101(Fe)/GO as a novel nanocarrier and investigation of its antimicrobial properties

**DOI:** 10.1039/d4ra90140a

**Published:** 2024-11-26

**Authors:** Fatemeh Shateran, Mohammad Ali Ghasemzadeh, Seyyed Soheil Aghaei

**Affiliations:** a Department of Chemistry, Islamic Azad University Qom Branch Qom I. R. Iran Ghasemzadeh@qom-iau.ac.ir; b Department of Microbiology, Islamic Azad University Qom Branch Qom I. R. Iran

## Abstract

Retraction of ‘Preparation of NiFe_2_O_4_@MIL-101(Fe)/GO as a novel nanocarrier and investigation of its antimicrobial properties’ by Fatemeh Shateran *et al.*, *RSC Adv.*, 2022, **12**, 7092–7102, https://doi.org/10.1039/D1RA08523A.

The Royal Society of Chemistry hereby wholly retracts this *RSC Advances* article due to concerns with the reliability of the data.

The SEM images in Fig. 4a, b and c are the same as other SEM images found in other publications, describing different materials. The XRD patterns in Fig. 5 have repeating segments between the traces.

Given the significance of these concerns, the findings presented in this paper are no longer reliable.

The authors were informed about the retraction of the article. Mohammad Ali Ghasemzadeh agreed with the decision, the other authors have not responded.

Signed: Mohammad Ali Ghasemzadeh

Date: 14th November 2024

Retraction endorsed by Laura Fisher, Executive Editor, *RSC Advances*

